# Self-isolation of an Italian long-term care facility during COVID-19 pandemic: A comparison study on care-related infectious episodes

**DOI:** 10.1515/med-2023-0822

**Published:** 2023-10-27

**Authors:** Noemi Venditti, Giulio Petronio Petronio, Melania Pinti, Giovanni Cutolo, Laura Pietrangelo, Laura Massini, Irene Magnifico, Marco Alfio Cutuli, Federica Petrone, Stefano Papini, Roberto Di Marco, Graziamaria Corbi

**Affiliations:** Department of Medicine, Health Science “V. Tiberio”, Università degli Studi del Molise, Via De Sanctis snc, Campobasso 86100, Italy; Istituto Dottrina Cristiana (Largo Istituto Dottrina Cristiana, 1, 67100 L’Aquila AQ), U.O. RSD e Casa di Riposo “Villa Santa Maria”, Montenero di Bisaccia (CB) 86036, Italy; Department of Translational Medical Sciences, University of Naples Federico II Napoli 80126, Italy; Italian Society of Gerontology and Geriatrics (SIGG), Campania Region section, Florence, Italy; UO Laboratorio Analisi, Responsible Research Hospital, Campobasso, Italy

**Keywords:** COVID-19/epidemiology, long term care facilities, self-isolation, antibiotic, infection, elderly, hospitalization/statistics, numerical data

## Abstract

The aim of this retrospective cohort study is to understand if and how much the preventive self-isolation approach might have been a valid model to avoid care-related infection, not only from COVID-19 but also from other non-viral infectious diseases. From March to May 2020, the healthcare and management staff of the Villa Santa Maria long-term care facilities, located in the village of Montenero di Bisaccia (Campobasso, Molise, Italy), decided to carry out a preventive self-isolation plan to safeguard the residents from SARS-CoV-2. The impact on other infectious diseases was evaluated by analyzing the antibiotic therapies prescription trend among the inpatients. Our data showed that although self-isolation protected residents and caregivers from SARS-CoV-2, it can also be associated with mobility reduction, leading to an increase in bedridden pathologies, namely, pressure ulcers and pressure sores. The simultaneous isolation of residents and caregivers in the same location significantly reduced any outside influence as a cause of possible infections.

## 1 Introduction

The newly discovered coronavirus is the cause of the infectious disease known as coronavirus disease (COVID-19). The majority of COVID 19 virus-infected individuals will experience mild to moderate respiratory disease and recover without the need for special care [1]. However, the disease has a more severe course in individuals with comorbidities such as hypertension, diabetes, cardiovascular disease, asthma, severe allergies, obesity, and chronic obstructive pulmonary disease [2]. Because of the higher prevalence of these conditions by aging, older adults are particularly affected by SARS-CoV-2, causing a more severe spectrum of clinical manifestations and higher mortality [3,4,5].

A 2020 study conducted by Yang et al. on 1,099 COVID-19 Chinese patients found that 15.1% of the infected subjects were 60 years old and above, and 27.0% presented severe symptoms [6]. This evidence has also been confirmed by *in vivo* animal studies where SARS-CoV-2 caused severe interstitial pneumonia with an increased viral replication in old monkeys’ lungs compared to young ones [7,8,9,10].

Elderly people who recovered from COVID-19 often experience sarcopenia, malnutrition, depression, and delirium [11]. The psychosocial impact added to the physical symptoms increased COVID-19’s long-term effects (also known as the post-COVID syndrome or Long COVID) [12].

Long-term care facility’s (LTCF) older residents were demonstrably the most at risk of adverse outcomes and mortality during the current pandemic [13].

The increase in mortality risk could be explained by the combination of a higher prevalence of comorbidities, especially chronic conditions, characterized by a low-grade inflammatory status [14,15], and the age-related immune and inflammatory responses decay [16,17]. These changes reduce the effectiveness of viral clearance, leading to an increase in cytokine release that ends with the “cytokine storm” [18]. According to recent immunological research, severe COVID-19 is characterized by an initial inability to inhibit the replication of the virus, followed by an uncontrolled hyperinflammatory response that damages the respiratory epithelium. The excessive production of various anti-viral and pro-inflammatory cytokines and chemokines results in the hyperinflammatory response, which causes immune cell recruitment, lung epithelial damage, microvascular thrombosis and hyperpermeability, as well as systemic inflammation with multiorgan damage [19].

Indeed, the high prevalence of functional and cognitive impairment and behavioral symptoms add to the risk posed to LTCF residents and environments that do not present barriers to infection control [20].

In this scenario, several government agencies (World Health Organization, the Centers for Disease Control, and the British Geriatrics Society) had drafted guidelines and statements concerning the prevention and management of COVID-19 in LTCF [21,22,23].

LTCF in Italy has been particularly affected by the COVID-19 pandemic. De Girolamo et al. [24] indicated that LTCF residents showed higher mortality rates than the community-dwelling elderly population. In addition, the risk of death among residents increased approximately four-fold during the pandemic compared with previous years (2016–2019) [24].

In the pandemic’s early stages, Italy suffered from a shortage of Personal protective equipment (PPE). Specifically, LTCFs were not prioritized in receiving new supplies. Workers and users were not sufficiently protected from the COVID-19 dissemination [25].

Following the outbreak of the global COVID-19 pandemic, from March 20 to 5 May 2020, the healthcare and management staff of the Villa Santa Maria LTCF, located in the village of Montenero di Bisaccia (Campobasso, Molise, Italy), decided to carry out a preventive self-isolation plan to safeguard the facility residents from SARS-CoV-2 infection.

The aim of this retrospective cohort study is to understand if and how much this preventive self-isolation might have been a valid experimental model to avoid care-related infectious episodes, not only from COVID-19 but also from other non-viral infectious diseases. Moreover, the effect of the isolation on antibiotic therapies (ATs) prescription was also checked.

## 2 Materials and methods

It was a retrospective observational study. The effect of the self-isolation was evaluated by comparing data on diseases and prescribed therapies, from March 20 to May 5, 2020, with those collected during the same period in the previous year (from March 20 to May 5, 2019). The latter group (2019) was used as a control

The study population consisted of the residents living at the LTCF “Villa Santa Maria” during both the 2019- and 2020-time frames under examination.

This report adheres to the consolidated standards for the reporting of longitudinal studies and was written according to the STROBE guidelines for Observational Studies in Epidemiology [26] (see the checklist in the Supporting Information)

At the beginning of the preventive isolation, 95 guests lived at the LTCF “Villa Santa Maria” located in a Southern Italy village, Montenero di Bisaccia (Campobasso, Molise Italy).

The patient care, feeding, and daily routines remained unchanged over the two periods under review, as well as the PPE used by caregivers. During the voluntary isolation period, for both residents and operators, the only change in routine care was the absence of contact with any other outstanding person.

In addition, as recommended by the Italian Ministry of Health, since January 22, 2020, all standard biosecurity measures and precautions have been taken to prevent airborne and contact transmission. In particular, masks, facial protection, non-sterile long-sleeve waterproof gowns, and gloves. In addition, FFP2-masks were used for procedures that might generate aerosols [27].

This is a retrospective observational cohort study from chart review and data have been collected solely from the medical record. As a result, there was no intervention and no interaction with the research subjects. All records were collected anonymously and under current Italian privacy laws (Legislative Decree no. 196 of 2003), with prior approval of the facility management. Ethics approval was not applicable for retrospective observational cohort study from chart review

The ATs, the type of antibiotic, and the pathology for which the AT was prescribed were examined. Moreover, additional information, such as age, existing diseases, use of devices and aids, and pre-existing conditions, were also taken into account to better evaluate the population characteristics.

### 2.1 Statistical analysis

Statistical analysis was performed by STATA 16 statistics software. Two-tailed *p* < 0.05 was considered statistically significant for all analyses. Categorical variables were summarized using frequencies and percentages; continuous variables were summarized using means and standard deviations since the distributions were symmetrical. For the univariate analysis, we used *χ* ^2^ tests to compare categorical variables between the groups and the Student’s t-test for independent samples to compare continuous variables between the groups.

## 3 Results

### 3.1 Study population

At the beginning of the preventive isolation, 95 guests lived at the LTCF. Twenty-four subjects were excluded because they were not present in the same period of the previous year 2019, which represented the term of comparison.

Then, the study population consisted of 71 residents living during both the time frames under examination at the LTCF.

As shown in Table 1, among the 71 subjects, 20 were men (28.17%), and 51 were women (71.83%). The mean age was 85.55 ± 9.94 years, with a median of 87. Only ten subjects were under 80 years old (14.08%), of whom 30% were men, with the remaining 70% being women.

**Table 1:** Residents attending the LTCF

**Total, *n* (%)**	**71 (100)**
**Gender,** ***n*** **(%)**	
Men	20 (28.17)
Women	51 (71.83)
**Age**	
Mean value ± SD	85.55 ± 9.94
Median	87
Under 80, *n* (%)	10 (14.08)
Men	3 (30.00)
Women	7 (70.00)
Autonomy in mobility, *n* (%)	19 (26.76)
Partial dysautonomy in mobility (using aids), *n* (%)	17 (23.94)
Total dysautonomy in mobility, *n* (%)	35 (49.29)
Incontinence, *n* (%)	62 (87.32)
Urinary	26 (41.93)
Uro-fecal	36 (58.06)
Fecal	0 (0.00)
Bladder catheter use, *n* (%)	14 (19.72)

The residents showed different degrees of self-sufficiency, intended as the capability to ambulate and continence. Thirty-five subjects had total dysautonomia in mobility (49.29%) requiring a wheelchair, while 17 were using walking aids (walkers or crutches), and only 19 subjects were fully independent in walking. Nine subjects were continent, compared to 62 incontinent (87.32%). The latter presented different degrees of incontinence, only urinary 26 (41.93%) and 36 (58.06%) uro-fecal.

Table 2 reports residents’ underlying diseases stratified by use or not of ATs. Sixteen (22.53%) out of the 71 subjects were on AT, i.e., 9 women and 7 men. The most common chronic diseases were grouped by apparatus or system.

**Table 2:** Residents and related underlying diseases with or without AT

	Total	AT 2019	No AT 2019	*p* value
Resident, *n* (%)	71 (100)	16 (22.54)	55 (77.46)	
**Gender, *n* (%)**				0.115
Men	20 (28.17)	7 (43.75)	13 (23.64)	
Women	51 (71.83)	9 (56.25)	42 (76.36)	
**Cardiovascular conditions,** ***n*** **(%)**				
Arterial hypertension	42 (59.15)	9 (56.25)	33 (60.00)	0.788
Cardiopathy	22 (30.98)	4 (25.00)	18 (32.73)	0.556
Arrhythmias including atrial fibrillation	12 (16.90)	2 (12.50)	10 (18.18)	0.594
PMK carriers	2 (2.82)	0 (0.00)	2 (3.64)	0.439
**Neurological conditions,** ***n*** **(%)**				
Cognitive impairment	20 (28.17)	5 (31.25)	15 (27.27)	0.756
Alzheimer’s disease (AD)	3 (4.23)	0 (0.00)	3 (5.45)	0.340
Dementia other than AD	12 (16.90)	3 (18.75)	9 (16.36)	0.823
Depressive-anxious status	9 (12.68)	0 (0.00)	9 (16.36)	0.083
Stroke with hemiparesis and motor aphasia	8 (11.27)	1 (6.25)	7 (12.73)	0.471
Psychiatric conditions	5 (7.04)	0 (0.00)	5 (9.09)	0.211
Parkinson’s disease	3 (4.22)	2 (12.50)	1 (1.82)	0.062
**Musculoskeletal conditions,** ***n*** **(%)**				
Femur Fractures	14 (19.72)	1 (6.25)	13 (23.64)	0.124
Ischial pubic fractures	3 (4.22)	0 (0.00)	3 (5.45)	0.340
Fractures – other sites	2 (2.82)	0 (0.00)	2 (3.64)	0.439
Previous fractures	1 (1.41)	0 (0.00)	1 (1.82)	0.587
Arthrosis	7 (9.86)	4 (25.00)	3 (5.45)	0.021
**Sensory conditions,** ***n*** **(%)**				
Hypoacusis	21 (29.58)	3 (18.75)	18 (32.72)	0.281
Hypovision	5 (7.04)	1 (6.25)	4 (7.27)	0.888
**Metabolic conditions,** ***n*** **(%)**				
Diabetes Mellitus Type II in OHD^1^ therapy	9 (12.68)	4 (25.00)	5 (9.09)	0.092
Diabetes Mellitus Type II in insulin therapy	3 (4.22)	1 (6.25)	2 (3.64)	0.647
Thyroidopathies	5 (7.04)	1 (6.25)	4 (7.27)	0.888
**Oncological conditions,** ***n*** **(%)**				
Previous pathologies	5 (7.04)	2 (12.50)	3 (5.45)	0.332
Ongoing pathologies	2 (2.82)	0 (0.00)	2 (3.64)	0.439
**Disease number,** ***n*** **(%)**				0.155
0 disease	1 (1.41)	1 (6.25)	0 (0.00)	
1 disease	7 (9.86)	1 (6.25)	6 (10.91)	
2 diseases	11 (15.49)	4 (25.00)	7 (12.73)	
≥3 diseases	52 (73.24)	10 (62.50)	42 (76.36)	

^1^OHD, Oral Hypoglycemic Drugs.

Data on comorbidity showed that most residents were affected by three or more pre-existing conditions (73.24%; Table 2). No statistically significant differences were found between the groups except for the higher prevalence of arthrosis (*p* = 0.021) in the AT group.

### 3.2 Effect of voluntary isolation (2020) on the prevalence of COVID-19 among residents.

During the 46 days voluntary isolation (from March 20 to May 5, 2020) periodic molecular swabs for COVID-19 screening were carried out by the Molise Department of Hygiene and Territorial Prevention on both residents and LTCF staff. No cases of SARS-CoV-2 infection were recorded during this period among the 71 LTCF residents and operators.

### 3.3 Effect of voluntary isolation (2020) on AT and non-viral infectious diseases

Table 3 shows the number of subjects with acute infections requiring AT during the two study periods. The voluntary isolation did not cause any significant change in the number of subjects requiring AT for bronchitis and cystitis recurrence. On the other hand, there was a reduction in tonsillitis (–2 cases from 2019 to 2020), with an increased number of acute non-previous bedridden conditions, namely, pressure ulcers and pressure sores (Table 3). Overall, a reduction of 31.25% in the number of subjects with acute infections requiring AT during voluntary isolation (2020) was observed.

**Table 3:** Number of subjects affected by acute infection requiring AT in 2019 and 2020

*n* (%)	2019 (*n* = 71)	2020 (*n* = 71)	Change	*p* value
Bronchitis	7 (9.86)	6 (8.45)	−1	0.559
Tonsillitis	2 (2.82)	0 (0.00)	−2	—
Cystitis	7 (9.86)	1 (1.41)	−6	0.739
Pressure ulcers	0 (0.00)	2 (2.82)	+2	—
Pressure sores	0 (0.00)	2 (2.82)	+2	—
Total acute infections	16 (22.54)	11 (15.49)	−5	0.232
Percentual change in number of subjects treated in 2020 vs 2019	−31.25%			

The antibiotic types used for the treatment of infectious diseases are shown in Table 4. A comparison of the 2020 data with the same period in the previous year (2019) showed no significant changes in the administration of amoxicillin, ceftriaxone, and ciprofloxacin. In 2019, the most used antibiotic drug was ciprofloxacin, as expected by the higher incidence of bronchitis in this period, while, in 2020, ceftriaxone was more frequently used. Overall, there was a 27.78% decrease in the number of antibiotics used in 2020 compared to 2019 (Table 4).

**Table 4:** Antibiotic prescription in 2019 and 2020

*n* (%)	2019 (*n* = 18)	2020 (*n* = 13)	Change	*p* value
Amoxicillin	4 (22.22)	2 (15.38)	−2	0.497
Ceftriaxone	5 (27.78)	5 (38.46)	0	0.862
Ciprofloxacin	7 (38.89)	2 (15.38)	−5	0.379
Fosfomycin	1 (5.55)	0 (0.00)	−1	—
Thiamphenicol	1 (5.55)	0 (0.00)	−1	—
Piperacillin	0 (0.00)	3 (23.08)	+3	—
Sulfamethoxazole/trimethoprim	0 (0.00)	1 (7.69)	+1	—
Total number of antibiotics used	18	13	−5	0.357
Percentual change in antibiotics prescribed in 2020 vs 2019	−27.78%			

AT duration (in days) for the two observation periods was analyzed for each antibiotic prescribed (Table 5). Despite Fosfomycin, thiamphenicol, and sulfamethoxazole/trimethoprim having the lowest mean durations, they were prescribed in only one of the periods studied (2019 for the first two and 2020 for the third, respectively, Table 4). The average days of piperacillin therapy in 2020 were the highest with a mean value of 23.3 ± 11.4 and a median value of 20 days. Regarding the three antibiotics prescribed in both study periods (i.e., amoxicillin, ceftriaxone, and ciprofloxacin), a significant difference was observed only for the latter (*p* = 0.0308, Table 5). On the other hand, treatment durations with amoxicillin and ceftriaxone remained almost unchanged in both observed periods (*p* = 0.117 and *p* = 0.900, respectively, Table 5).

**Table 5:** AT duration (days) in 2019 and 2020

Year	2019	2020	*p* value
**Fosfomycin, days**			
Range (min–max),	0–2	—	
Mean value +/− SD	2.00 ± 0.00	0	—
Median	2	0	
**Thiamphenicol, days**			
Range (min–max)	0–6	—	
Mean value +/− SD	6.00 ± 0.00	0	—
Median	6	0	
**Sulfamethoxazole/trimethoprim, days**			
Range (min–max)	—	0–3	
Mean value +/− SD	0	3.00 ± 0.00	—
Median	0	3	
**Piperacillin, days**			
Range (min–max)	—	14–36	—
Mean value +/− SD	0	23.3 ± 11.4	—
Median	0	20	—
**Amoxicillin, days**			
Range (min–max)	4–8	0–4	
Mean value +/− SD	6 ± 1.41	4 ± 0.00	0.117
Median	6	4	
**Ceftriaxone, days**			
Range (min–max)	6–12	5–14	
Mean value +/− SD	7.6 ± 2.61	7.83 ± 3.25	0.900
Median	6	7	
**Ciprofloxacin, days**			
Range (min–max)	5–6	6–16	
Mean value +/− SD	5.75 ± 0.46	11 ± 7.07	0.0308
Median	6	11	

Finally, the prescribed antibiotics and the pathologies diagnosed in the two study periods were analyzed (Table 6). For amoxicillin, always prescribed in monotherapy, a reduction in case numbers was observed in 2020 compared to 2019. In the first period, this antibiotic was used for bronchitis, cystitis, and tonsillitis treatment (6 cases total), while in the second one, only for two cases of pressure ulcers. Ceftriaxone’s prescription remains unchanged, and it was used for bronchitis treatment. Ciprofloxacin was used in 2019 for five cystitis cases, in four cases in mono and one in poly AT, while in 2020, this drug was adopted in one case of pressure sore in association with other AT. On the other hand, bronchitis treatment with ciprofloxacin remained unchanged in both periods. Fosfomycin and thiamphenicol were prescribed for cystitis and bronchitis, respectively, in 2019 only in association with other antibiotics, as well as piperacillin and sulfamethoxazole/trimethoprim that was prescribed for bronchitis in polytherapy, pressure sores, and cystitis in monotherapy only during 2020.

**Table 6:** Acute infection requiring AT and antibiotic prescribed in 2019 and 2020

Antibiotic	2019			2020		
	Acute infection requiring AT	Mono/Poly AT	Case number	Acute infection requiring AT	Mono/Poly AT	Case number
Amoxicillin	Bronchitis	Mono	2	Pressure ulcers	Mono	2
	Cystitis	Mono	2			
	Tonsillitis	Mono	2			
Ceftriaxone	Bronchitis	Mono	2	Bronchitis	Mono	3
					Poly	2
		Poly	3			
Ciprofloxacin	Bronchitis	Poly	2	Bronchitis	Poly	1
	Cystitis	Mono	4	Pressure sores	Poly	1
		Poly	1			
Fosfomycin	Cystitis	Poly	1	—		
Thiamphenicol	Bronchitis	Poly	1	—		
Piperacillin	—			Bronchitis	Poly	2
				Pressure sores	Mono	1
Sulfamethoxazole/trimethoprim	—			Cystitis	Mono	1

## 4 Discussion

Lockdowns and additional restrictive measures were adopted worldwide to reduce the pandemic impact on healthcare. Containment measures that can reduce the infection spread include isolating infected residents in single rooms and limiting social activities. This latter solution may not be practicable in many LTCFs with limited housing capacity, so the infected are often grouped as an alternative to single isolation [28].

Long-term preventive self-isolation measures implemented in LTCFs during the COVID-19 pandemic can have significant implications for the health and well-being of the residents, although these measures are intended to mitigate the spread of the virus, they can give rise to various health challenges for the elderly population residing in these facilities. The potential long-term effect on residents must be acknowledged and mitigated through a holistic and compassionate approach that considers both physical and mental aspects [29].

Even before the pandemic, the effects of social isolation and loneliness on LTCFs residents were well known, especially for those most at risk. Although it is difficult to assess their impact on residents’ life quality, the introduction of social distancing policies may cause harm to this vulnerable population [30,31].

As expected, COVID-19 mortality increased significantly in LTCFs. In Europe, 30–70% of all deaths occurred among nursing home residents [32]. In 2020, 2 months observational study conducted in an Italian nursing home revealed an increase in mortality of 40%, compared to 6.4% in the previous year [33].

In this scenario, the 46 days of voluntary preventive self-isolation carried out by the health care and management staff of the LTCF “Villa Santa Maria,” protected residents and caregivers from SARS-CoV-2 infection and ensured elderly well-being (social activities and accommodation were not altered).

Special remarks must be made about infectious diseases in older people compared to younger individuals. Indeed, diseases are relatively more frequent and severe due to pre-existing conditions which contribute to increased death rates in the elderly [34]. Among these, advanced age together with cardiovascular, neurological, musculoskeletal, skin lesions, sensory deficits, metabolic, oncological, and respiratory pathologies are the most relevant.

The population under investigation had many risk factors for infection disease: the median age of 87 years, high prevalence of total dysautonomia in mobility (49.29%), and the occurrence of three or more previous diseases (73.24%) were the main ones. According to the literature, the most frequent treatment indications for ATs were Respiratory tract infections (RTIs), Urinary tract infections (UTIs), and Skin and soft tissue infections (SSTIs). On the other hand, the most often prescribed antibiotics were amoxicillin and penicillin-class drugs, cephalosporins, and fluoroquinolones [35,36,37,38]. These data are in agreement with those reported for the study population in both periods (Tables 3 and 4).

Penicillin is often chosen to treat susceptible pathogens infecting lungs, urinary tract, skin and soft tissues, bones and joints, gastrointestinal tract, and inflamed meninges. In 2019, amoxicillin was used in six cases (Table 6). On the other hand, in 2020, the number of prescriptions was reduced by one-third (Table 6). In the same way, the ureidopenicillin piperacillin was prescribed only in 2020 for three cases (Table 6), due to its extended-spectrum activity [39].

Cephalosporins, prescribed for bronchitis in both periods (Table 6), belong to the β-lactam antibiotics with favorable pharmacokinetics, relative safety, efficacy, and a broad antimicrobial spectrum; for these reasons, they are often recommended for the treatment of the infection in the elderly [40]. In an epidemiological survey on the incidence and treatment of Community-acquired pneumonia (CAP) in Italy conducted in 2006 by Viegi et al. among 699 subjects with suspected CAP, cephalosporins were prescribed in more than a quarter of cases (27.1%) [41]. According to the authors, these findings agree with previous epidemiological data showing that third-generation cephalosporins are the most frequent prescriptions in initial antibiotic therapy for community-acquired lower RTI in Italy [42].

Quinolones are helpful in the treatment of complicated UTIs, bacterial prostatitis, SSTIs, pneumonia, malignant otitis externa, and bacterial diarrhea caused by susceptible pathogens [43].

The largest differences in AT duration (days) were observed in this scenario for ciprofloxacin (i.e., from 5.75 ± 0.46 to 11 ± 7.07 days, *p* 0.0308; Table 5). This increase was due to the treatment of a bronchitis case (16 days) in 2020 (Tables 5 and 6). Along with bronchitis, ciprofloxacin was used to treat cystitis and pressure sores in 2019 and 2020, respectively (Table 6).

Fosfomycin, used for one case of cystitis in 2019 (Table 6), is a broad-spectrum drug with excellent bactericidal activity against Gram-positive and negative microorganisms. It is considered the first-choice oral drug for uncomplicated UTI caused by Gram-negative bacteria [44]. Due to its pharmacokinetic characteristics, dosage adjustments are not required in elderly patients, pregnant patients, or in cases of renal or hepatic insufficiency [45].

Thiamphenicol, prescribed for one case of bronchitis in 2019 (Table 6), is a broad-spectrum bacteriostatic antimicrobial agent indicated against certain Gram-positive cocci, which can exert a bactericidal effect at 2–4 times the minimum inhibitory concentration. Due to its pharmacokinetic properties, it is quickly absorbed orally, parenterally, and by aerosol. One of its derivates, the acetylcysteine glycinate ester, is particularly indicated in the treatment of RTIs with dense or mucopurulent secretions. Indeed, after parenteral or aerosol administration, it is hydrolyzed in the lungs with rapid release of thiamphenicol and N-acetylcysteine (NAC). The mucolytic activity of NAC and its ability to inhibit bacterial adhesion facilitates the removal of secretions and the elimination of pathogens from the epithelial cells of the respiratory tract [46].

Together with Fosfomycin, the trimethoprim/sulfamethoxazole combination, prescribed for one cystitis case in 2020, is most commonly used to treat uncomplicated UTIs. Due to its tolerability, the spectrum of activity against suspected uropathogens and favorable pharmacokinetic profiles make it suitable for administration to the elderly. Indeed, this drug concentrates more in urine rather than serum, ensuring greater efficacy. In addition, the strong action against Gram-negative aerobic strains and limited interference on the anaerobic vaginal and fecal microbiota appears to provide a better long-term outcome [47].

This is the first study providing information on the prevalence of non-viral infectious episodes and their incidence in elderly LTCF residents under ATs by using a self-isolation model. The simultaneous isolation of residents and caregivers in the same location significantly reduced any outside influence as a cause of possible infections. Also, the investigated population, being the same in both periods, ensures the consistency of the two analyzed groups.

Nevertheless, as a retrospective chart review, some limitations regarding sampling must be pointed out. The population was small (only 16 and 11 out of 71 subjects in AT in 2019 and 2020, respectively), and only some medical information was available. Moreover, the unavailability of microbiological characterization did not allow a more in-depth analysis of infection epidemiology in LTCFs

To sum up, the observed infection reduction trend is not significant due to the sample’s small size and the subject’s lower exposure to etiological agents from outside. Even though our data showed that self-isolation could be associated with subject mobility reduction, and increasing bedridden pathologies, namely, pressure ulcers and pressure sores (Table 3), there are also benefits from self-isolation in LTCFs as a preventive measure. These include infection control, protection of residents, prevention of healthcare system overload, and staff transmission [48]. Despite these advantages, there are also some implications and limitations that affect both residents and caregivers, thus implementing self-isolation measures can strain LTCF staff, who may face increased workload and burnout, impacting the quality of care provided to residents. Particular relevance is also the ethical considerations, balancing infection control measures with the rights and well-being of residents raises ethical dilemmas that require careful consideration [49].

Self-isolation measures in LTCFs have the potential to prevent infectious disease outbreaks and protect vulnerable residents. However, these measures also come with a range of implications and limitations, including adverse effects on mental health, cognitive well-being, and quality of life. It is essential for LTCFs to balance infection control efforts with strategies to address the potential negative impacts and ensure the overall well-being of their resident. Therefore, it is essential to balance infection control efforts with strategies to address the potential negative impacts and ensure the overall well-being of their residents.

Finally, more studies should be planned to better evaluate the performance of this model over a more extended period (more than 46 days), recruiting a larger population

belonging to different nursing homes. This would provide a better understanding of the role played by other factors, such as intraindividual variability in different communities and the management of infectious diseases in LTCFs.

## Supplementary Material

Supplementary Table
